# Research Progress on Mitochondrial Dysfunction in Diabetic Retinopathy

**DOI:** 10.3390/antiox11112250

**Published:** 2022-11-15

**Authors:** Yiwei Wu, Haidong Zou

**Affiliations:** 1 Shanghai Jiao Tong University School of Medicine, Shanghai 200025, China; 2 Department of Ophthalmology, Shanghai General Hospital, Shanghai Jiao Tong University School of Medicine, Shanghai 200080, China

**Keywords:** diabetic retinopathy, mitochondrial, metabolism, epigenetics, mitophagy, apoptosis

## Abstract

Diabetic Retinopathy (DR) is one of the most important microvascular complications of diabetes mellitus, which can lead to blindness in severe cases. Mitochondria are energy-producing organelles in eukaryotic cells, which participate in metabolism and signal transduction, and regulate cell growth, differentiation, aging, and death. Metabolic changes of retinal cells and epigenetic changes of mitochondria-related genes under high glucose can lead to mitochondrial dysfunction and induce mitochondrial pathway apoptosis. In addition, mitophagy and mitochondrial dynamics also change adaptively. These mechanisms may be related to the occurrence and progression of DR, and also provide valuable clues for the prevention and treatment of DR. This article reviews the mechanism of DR induced by mitochondrial dysfunction, and the prospects for related treatment.

## 1. Introduction

DR is one of the common complications of diabetes mellitus, which can lead to proliferative retinopathy, macular edema, and eventually blindness [1]. The retina consists of photoreceptor cells, horizontal cells, bipolar cells, amacrine cells, Müller cells, and ganglion cells. Embedded in the layered structure are astrocytes, microglial cells, and the retinal vasculature, which is composed of endothelial cells and pericytes. These cells play an important role in the homeostasis of the retina [2]. In diabetic patients, long-term hyperglycemia can cause damage to these cells, and microvascular endothelial cells injury can lead to pathological changes such as vascular exudation [3], microaneurysm formation [4] and thrombosis [5]. The damage of cone cells and glial cells affects the generation and conduction of vision and destroys the blood-retinal barrier [6]. The mechanism of cell damage under a high glucose environment may be related to oxidative stress [7], metabolic abnormalities [8], epigenetic modification [9], etc., while mitochondrial dysfunction induced by high glucose, as a well-known mechanism, has been widely studied. This article reviews the mechanism of diabetic retinopathy induced by mitochondrial dysfunction.

## 2. Overview of Mitochondria

Mitochondria are energy-producing organelles in eukaryotic cells, which participate in metabolism and signal transduction, and regulate cell growth, differentiation, aging and death, have been widely studied in the field of biology and medicine.

Mitochondria are an important site for the metabolism of the three major nutrients in the cell, and the tricarboxylic acid cycle (TCA) in the mitochondrial matrix is the common hub of sugar metabolism and lipid metabolism. The energy in the saccharide and the lipid molecules is transmitted in the form of protons and electrons through Nicotinamide adenine dinucleotide (NADH) and reduced flavin adenine dinucleotide (FADH2) to the electron transport chain (ETC) on the inner mitochondrial membrane. Protons and electrons are finally transferred to oxygen through complexes I-IV in the ETC (including coenzyme Q and a variety of cytochromes) to produce H_2_O and a large number of adenosine triphosphates (ATPs). Mitochondria have a bilayer phospholipid membrane structure, and the molecule transport across the mitochondrial membrane undergoes a strict “quality control” mechanism, which ensures the relative stability of the mitochondrial matrix environment and the intermembrane space environment, so as to ensure the normal metabolism [10].

Unlike other organelles, mitochondria have their own separate set of genomes, namely mitochondrial DNA (mtDNA). Mitochondrial proteins are partially encoded by nuclear genes and transported from the cytoplasm to the mitochondria across the membrane, while the other part is directly transcribed and expressed by mtDNA. This pattern reflects the process of biological evolution, and also illuminates that the expression, modification, and assembly of mitochondrial proteins are strictly and complexly regulated [11,12].

Mitochondria in cells undergo dynamic changes. The mechanism of mitochondrial fusion and fission is essential to maintain its normal morphology, quantity, distribution, and function, which can resist cell aging and play a compensatory or remedial role under stress conditions [13]. Damaged or dysfunctional mitochondria can disrupt cellular homeostasis [14], and PINK1/Parkin-mediated mitophagy can clear these mitochondria [15], exerting mitochondrial quality control and cytoprotective effects.

However, the number, size, structure and physiological functions of mitochondria may change under pathological conditions such as ischemia and hypoxia, nutrient deficiency or imbalance, endotoxin injury, calcium overload, and so on. ETC dysfunction and (or) antioxidant deficiency can lead to oxidative stress [16,17], producing excessive ROS, damaging cell structure and interfering with normal metabolism [3], while repressing ATP production [18,19]. Oxidative stress and calcium overload induce mitochondrial permeability transition (mPT) mediated by mitochondrial permeability transition pore (mPTP), which leads to the extravasation of mitochondrial contents such as cytochrome C, causing apoptosis and inflammatory response [20]. Prolonged opening of mPTP can lead to NADH consumption, damage complex I, and further aggravate oxidative stress [21,22]. On the basis of these processes, mtDNA can be damaged, and the mitochondrial dynamic regulation and mitophagy mechanism will also be destroyed [23], further affecting mitochondrial homeostasis and resulting in corresponding pathological changes in cells and tissues.

In a long-term hyperglycemic environment, the above mitochondria-related pathological processes may exist in retinal cells, causing damage to various types of retinal cells over time, leading to DR.

## 3. Mitochondrial Dysfunction Secondary to Diabetes Mellitus Induces Retinopathy

### 3.1. Metabolic Changes

#### 3.1.1. Glucose Metabolism

Under the stress of hyperglycemia, the nerve cells, pigment epithelial cells, and capillary endothelial cells in the retina can undergo glycometabolic reprogramming. Dysfunction of the ETC, as the core change, results in energy generation disturbance [24], oxidative stress, abnormal glucose metabolite production, retinal microcirculation disorders, and other pathophysiological changes, inducing the occurrence of retinopathy [25].

In the case of continuous hyperglycemia, the overload of NADH and FADH2 produced by TCA can induce high mitochondrial membrane potential, which makes the ETC stagnate in complex III, and the electrons and protons carried by coenzyme Q are difficult to transfer to the downstream of the respiratory chain. At this time, oxygen molecules as electron acceptors can generate superoxide, mediating changes in cell metabolism [26].

- The activity of GAPDH is reduced, activating the polyol pathway

Excess accumulation of intracellular superoxide activates PARP with the depletion of nicotinamide adenine dinucleotide (NAD^+^) [27]. As a result, the activity of glyceraldehyde-3-phosphate dehydrogenase (GAPDH) is significantly reduced [28], and the glycolysis process is inhibited. At this time, the polyol pathway of glucose metabolism is over-activated; in other words, glucose is converted to sorbitol under the action of aldose reductase, and then sorbitol can be oxidized to fructose [29]. Due to the decrease of the NAD^+^/NADH ratio, sorbitol, the intermediate product of polyol pathway, accumulates, and its high hydrophilicity can lead to cell hypertonicity, damage retinal capillary endothelial cells, and cause micro-circulation disorders [30]. The activation of the polyol pathway is also accompanied by further depletion of NAD^+^, which has a positive feedback effect on oxidative stress [31]. The C106T polymorphism in the aldose reductase gene is associated with the severity of retinopathy in type 2 diabetes [32]. Inhibition of aldose reductase reduces neuronal apoptosis, glial response, and complement deposition and retinal ne-ovascularization in DR [33].

- The inhibition of glycolysis also leads to overactivation of the hexosamine pathway

The inhibition of glycolysis also promotes the metabolism of fructose-6-phosphate via the hexosamine pathway to UDP-*N*-acetyl glucosamine (UDP-GlcNAc), which promotes the synthesis of proteoglycans and *O*-linked glycoproteins. *O*-GlcNAc can covalently modify the transcription factor Sp1, which activates the expression of glucose-responsive gene plasminogen activator inhibitor-1 (Pal-1) in vascular smooth muscle cells [34], thus promoting DR [35]. It has been found that the level of hexosamine in the retinal tissue of diabetic patients is increased [30], which promotes the downstream *O*-GlcNAc to play a role in signal transduction, transcription regulation, regulation of cytoskeletal dynamics, and cell division [36]. Glucosamine, a product of the hexosamine metabolic pathway, causes retinal pericyte loss and the formation of acellular capillaries in non-diabetic animals by inhibiting VEGFR2 and Ang2 in the normal retina [37]. High hexosamine levels can also enhance cellular oxidative stress by positive feedback, further damaging the mitochondrial respiratory chain by promoting ROS generation [38]. However, oral administration of glucosamine protected retinal neurons in a mouse model of DR. The mechanism of this bidirectional regulation remains to be clarified [37].

- Triose phosphate accumulates, activating several PKC-related pathways

Attenuation of GAPDH activity results in increased concentration of intracellular triose phosphate, which can be decomposed and acylated to diacyl glycerol(DAG) [39], and also participates in the formation of various advanced glycation end products (AGEs) [40], all of which play an important role in the pathogenesis of DR. On the one hand, DAG can directly activate protein kinase C (PKC) in the retina, mainly β- and δ-type isozymes, while the activation of PKC-α and -ɛ is also found in the retina of diabetic rats [39]. On the other hand, AGE-mediated signaling pathways as well as metabolic products of the polyol pathway are also associated with PKC activation [41,42]. Activated PKC increases the activity of cytosolic phospholipase A2 and promotes the production of arachidonic acid and prostaglandin E2 (PGE2); the latter inhibits the activity of Na+/K+ ATPase [43], leading to cell edema. PKC-α is related to the increased permeability of vascular endothelial cells under the condition of high glucose [44], and the activation of PKC-β can mediate retinal vasoconstriction and blood flow reduction by inhibiting the production of nitric oxide and increasing the activity of endothelin-1 [45]. These mechanisms may result in the injury and necrosis of retinal cells and affect the function of the retina. In addition, PKC can induce the expression of vascular endothelial growth factor (VEGF) in retinal tissue, thus increasing vascular permeability and promoting angiogenesis, which is closely related to the non-proliferative and proliferative changes in DR, respectively [46,47].

- Massive synthesis of AGEs exerts pathological effects inside and outside cells

AGEs refer to a series of proteins, of which amino-groups are modified by intracellular dicarbonyl products (including glyoxal, methylglyoxal, and 3-deoxyglucosone), can be produced massively and secreted to extracellular medium when glycolysis is blocked. AGEs can change the function of intracellular and extracellular proteins, and are associated with intracellular signal transduction, metabolic regulation and cell adhesion [26]. Stitt et al. found that the content of AGEs in retinal blood vessels of diabetic mice was increased [48], and AGEs could interact with the receptor for advanced glycation end products (RAGE) on the plasma membrane of adjacent cells, exerting pathological effects, including promoting the activation of nuclear factor Kappa B (NF-κB) in retinal pericytes to induce their apoptosis, up-regulating the expression of VEGF in retinal capillary endothelial cells to increase vascular permeability, and activating the RhoA/ROCK signaling pathway as well as inducing moesin phosphorylation to promote retinal neovascularization [49,50,51]. AGEs can also lead to retinal vascular hyperpermeability by disrupting intercellular adhesion and tight junctions [52]. AGEs can up-regulate the expression of intercellular adhesion molecule-1 (ICAM-1) on the surface of endothelial cells, resulting in leukocyte stasis in the microcirculation and causing microcirculation disorders [53]. Ying et al. proposed that the severity of DR can be predicted by measuring AGEs [54]. As a result, researchers have begun to explore the possibility of delaying the progression of DR by inhibiting AGEs. Hammes et al. found that AGE-inhibitor amino-guanidine can inhibit the proliferation of abnormal retinal endothelial cells and significantly reduce pericyte shedding, thereby inhibiting the progression of DR [55]. Endogenous Glyoxalase I can inhibit the production of AGEs, and Maisonpierr et al. have found that its overexpression could inhibit the increase of Angiopoietin-2 (Ang-2) expression in Müller cells induced by hyperglycemia, thereby reducing damage to pericytes and capillary endothelial cells [56].

**Figure 1:**
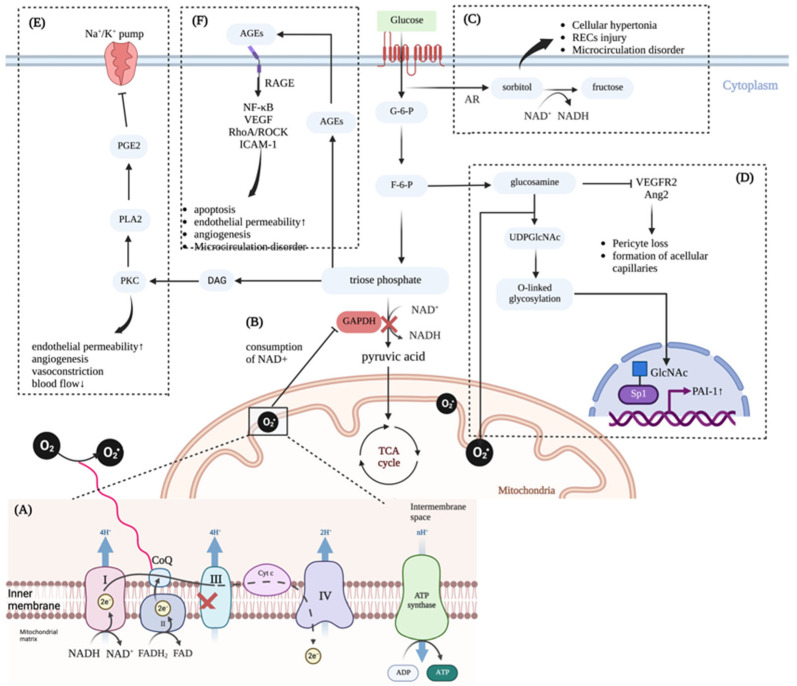
(**A**) Under the condition of high glucose, the overload of NADH and FADH2 produced by the TCA cycle in retinal cells can induce high mitochondrial membrane potential, which makes the ETC stagnate in complex III, and the electrons and protons accepted by CoQ are difficult to transfer to the downstream. At this time, O_2_ can be used as electron acceptors to generate superoxide. (**B**) The accumulation of intracellular superoxide can consume NAD^+^, thus inhibiting the activity of GAPDH and blocking the glycolytic pathway. (**C**) The polyol pathway of glycolysis is activated, that is, glucose is converted into sorbitol under the action of aldose reductase, and sorbitol can be catalyzed by NAD^+^ into fructose. Due to the consumption of NAD^+^, sorbitol accumulates, leading to cell hypertonicity, damaging retinal capillary endothelial cells, and causing microcirculation disorders. (**D**) The metabolic pathway of hexosamine is activated. The produced glucosamine can lead to the loss of retinal pericytes and the formation of acellular capillaries by inhibiting VEGFR2 and Ang2 in the normal retina. At the same time, the production of UDPGlcNAc increases, promoting the synthesis of *O*-linked glycoprotein. The latter covalently modifies transcription factor Sp1, activating the expression of glucose-responsive gene Pal-1 in vascular smooth muscle cells, thus promoting the occurrence of DR. (**E**) The decrease of GAPDH activity leads to the increase of triose phosphate, which can produce DAG and also participate in the formation of various AGEs. DAG can directly activate PKC, increasing the activity of PLA2 and promoting the production of PGE2, thereby inhibiting the activity of Na+/K+ ATPase. PKC itself is associated with increased permeability of retinal vascular endothelial cells, angiogenesis, vasoconstriction, and decreased blood flow. (**F**) AGEs can interact with RAGE to promote the activation of NF-κB, upregulate the expression of VEGF and ICAM-1, and activate the RhoA/ROCK signaling pathway, thus promoting apoptosis, increasing vascular permeability, causing microcirculation disorders, and inducing angiogenesis in the retina. Abbreviations: NADH, nicotinamide adenine dinucleotide; FADH2, flavine adenine dinucleotide, reduced; TCA cycle, tricarboxylic acid cycle; ETC, electron transfer chain; CoQ, coenzyme Q; NAD^+^, nicotinamide adenine dinucleotide; VEGFR2, vascular endothelial growth factor receptor 2; Ang2, angiopoietin-2; UDPGlcNAc, uridine diphosphate-*N*-acetylhexosamine; Pal-1, plasminogen activator inhibitor-1; GAPDH, glyceraldehyde-3-phosphate dehydrogenase; DAG, diacylglycerol; PKC, protein kinase C; PLA2, phospholipase A2; PGE2, prostaglandin E2; AGE, advanced glycation end products; RAGE, receptor for advanced glycation end products; NF-κB, nuclear factor kappa-B; VEGF, vascular endothelial growth factor; ICAM-1, intercellular cell adhesion molecule-1; Cyt c, cytochrome c; G-6-P, glucose-6-phosphate; F-6-P, fructose-6-phosphate. The figure is created with biorender.com (https://biorender.com/, accessed on 2 October 2022).

To sum up, the reprogramming of glucose metabolism, the activation of PKC, and the production of AGEs may all contribute to the occurrence of DR (Figure 1), while the specific mechanism remains to be clarified. It is worth mentioning that manganese superoxide dismutase (MnSOD) and Uncoupling Protein 1 (UCP-1) can inhibit almost all of the above cell biological changes to varying degrees [26], proving that oxidative stress plays a central role in the process of glucose metabolism disorder and retinopathy.

#### 3.1.2. Lipid Metabolism

Abnormal lipid metabolism occurs in more than 75% of individuals with type 2 diabetes [57]. Excessive lipid accumulation and abnormal lipid metabolites have been proved to be important mechanisms for the progression of DR. Mitochondria, as the site of lipid metabolism, incur damage related to abnormal lipid metabolism [58]. High concentration of palmitate culture and high glucose induction can play a synergistic role in retinal endothelial cells, aggravating mtDNA damage [59]. Excessive production of ceramide, acrolein and incomplete β-oxidation products of fatty acids in retinal cells under high glucose environment are also associated with mitochondrial damage.

Under physiological conditions, activated fatty acids generate acyl-CoA, which enters mitochondria for β-oxidation, followed by terminal metabolism through the Krebs cycle [60]. In mice with DR, the β-oxidation of fatty acids in the mitochondria of retinal cells is incomplete, and excessive intermediate products accumulate to produce toxic lipid peroxidation products, which in turn cause mitochondrial damage [61,62].

- Ceramide accumulation causes ETC disorder and mitochondrial pathway apoptosis

There are many kinds of sphingolipids in mitochondria, including sphingomyelin and ceramide [63,64], as well as metabolism-related enzymes, such as ceramide synthetase, acidic and neutral sphingomyelinase, and neutral ceramidase [58]. In normal cells, ceramide is minimally expressed in the cytoplasmic membrane, while a high glucose level induces ceramide accumulation in a concentration- and time-dependent manner [65,66,67]. Levitsky et al. found that ceramide induced by acid sphingomyelinase increased in mitochondria of retinal pigment epithelial cells in streptozotocin-induced diabetic rats, resulting in respiratory chain dysfunction. Inhibition of acid sphingomyelinase restores the function of the respiratory chain [68]. In terms of mechanism, excessive ceramide can inhibit the electron transfer function of complex III [69], promote the production of Sphingosine-1-Phosphate (S1P) and hexadecenal and the activation of BAX/BAK, increasing the permeability of the mitochondrial outer membrane and the release of cytochrome C. Finally, apoptosis is induced [70,71].

- Acrolein overproduction promotes oxidative stress, causing mitochondrial damage

Acrolein is overproduced in the retinal cells of diabetic individuals, and the production of its protein-bound products is associated with the progression of DR [72]. As a biomarker, FDP-lysine can reflect the content of acrolein, which is increased in hemoglobin and vitreous humor in patients with proliferative diabetic retinopathy [73,74], and its level in retinal Müller cells of diabetic animal models also parallels the progress of the disease [75]. Polyamine oxidation and lipid peroxidation are the main intrinsic pathways of acrolein generation during DR pathology [72,76]. By depleting antioxidants such as glutathione [77] and promoting oxidative stress by forming protein carbonyls [78], excessive acrolein reduces the membrane potential of mitochondria and the activity of complexes I, II, and IV, resulting in mitochondrial damage in retinal pigment epithelial cells [79,80]. The corresponding metabolic and biochemical changes also occur in pathological processes such as retinal inflammatory response, ganglion cell degeneration, blood-retinal barrier damage, and Müller cell dysfunction [72].

- Extracellular accumulation of modified lipoprotein induces apoptosis

Extracellular accumulation of abnormal lipid metabolites can also promote DR. Highly oxidized and glycosylated low-density lipoprotein accumulation in the lumen and extravasation of retinal capillaries is an early feature of DR [81]. Abnormally modified lipoproteins can induce apoptosis by increasing mitochondrial outer membrane permeability through the Bax pathway [82].

### 3.2. Epigenetic Changes

High glucose can not only reprogramme the metabolism of cells, but also regulate epigenetics by changing the activity of corresponding modifying enzymes, resulting in changes in gene expression, including a number of nuclear-encoded mitochondrial function-related genes and mitochondrial genome genes. The reprogramming of the expression of these genes is closely related to mitochondrial damage.

The most prominent epigenetic change is increased methylation levels [9]. Among patients with type II diabetes, DNA methylation of key genes in islets increases, which inhibits their expression [83,84,85,86], and DNA methylation levels are altered in adipose tissue, liver, and skeletal muscle [87,88,89,90,91,92]. Methylation of these genes may be directly induced by high glucose and HbA1C [85,86,93]. Kowluru et al. found that in the rat model of type 2 diabetes induced by high-fat diet, DNA Methyltransferase 1 (DNMT1) in retinal capillaries was highly expressed in the early stage of diabetes, which could affect the methylation of a series of genes related to retinal damage [94].

Ras-related C3 botulinum toxin substrate 1 (Rac1) is a component of the NADPH oxidase 2 (Nox2) holoenzyme [95], and the latter is able to induce ROS production in mitochondria [96], mediating oxidative stress. In the rat model of type 2 diabetes mellitus induced by a high-fat diet, the Rac1 promoter was methylated in retinal microvascular endothelial cells, and a large number of Nox2 produced promoted the accumulation of intracellular ROS, resulting in the damage to mtDNA and the respiratory chain [94]. In retinal cells of DR patients, methylation of H3K4 in the Keap1 promoter was significantly increased, which activated Keap1 transcription, blocking the nuclear transport of nuclear factor erythroid-2 related factor 2 (Nrf2), thereby inhibiting its antioxidant effects and exacerbating mitochondrial damage [97,98,99]. Histone hypermethylation occurred in Sod2 promoter under high glucose conditions (H3K4 monomethylation and dimethylation decreased, while trimethylation increased), accompanied by an increase in acetylation level (H3K9ac). As a result, the down-regulation of Sod2 expression and the reduction of intracellular antioxidant MnSOD content also promote oxidative stress damage [100,101,102]. Mitofusin 2 (Mfn2), which mediates mitochondrial outer membrane fusion, is down-regulated in retinal endothelial cells cultured in high glucose due to hypermethylation of the promoter, interfering with mitochondrial homeostasis [103].

Mitochondrial DNA (mtDNA), which does not bind to histones, encodes a total of 13 proteins, all of which are important components of the respiratory chain. DNA polymerase γ, encoded by the nuclear gene POLG, is responsible for mtDNA replication. In DR, the CpG island of POLG regulatory region is highly methylated, which inhibits the transcription of POLG and affects the replication of mtDNA [104]. MutL homolog 1 (MLH1) is involved in mismatch repair during mtDNA replication. In human retinal endothelial cells cultured with high glucose, the methylation level of MLH1 promoter is increased, down-regulating its transcription and affecting the replication accuracy and function of mtDNA [105], which may reduce the activity of respiratory chain complex I and the antioxidant capacity of mitochondria [106].

The mtDNA itself is also susceptible to methylation under high glucose conditions. This mostly occurs in the Displacement loop (D-Loop) of mtDNA, which, as a non-coding region, contains transcription elements and also controls DNA polymerase γ-dependent mtDNA replication. D-Loop has a loose structure and is susceptible to various modifying enzymes [107]. Mitochondrial DNA methyltransferase (DNMT) is also highly expressed in retinal cells under high glucose condition, which can highly methylate the D-Loop region of mtDNA and cause mitochondrial damage in retinal cells [108,109]. Inhibition of DNMT can reduce mtDNA damage, improve transcriptional repression of mtDNA induced by high glucose, and restore ETC function [110]. At the same time, inhibition of promoter methylation may restore the activity of Sod2 (see above), which can inhibit the methylation of D-Loop in mouse retinal microvascular endothelial cells and ensure the operation of base mismatch repair mechanism [111].

Interestingly, Zhong et al. found that the promoter methylation level (H3K9me2) of Matrix Metalloproteinase-9 (MMP-9) in retinal microvascular endothelial cells of diabetes mice decreased, while the acetylation level (H3K9Ac) increased [99,112,113,114], resulting in high expression of MMP-9, damaging mitochondria and inducing apoptosis of retinal microvascular cells [112,115]. In view of this, the influence of a high glucose environment on epigenetic modification of the genome may be diverse and complex. More studies are needed to clarify the expression patterns of mitochondria-related nuclear coding genes and mitochondrial genome in retinal cells under high glucose environment, and the exploration of omics can also provide valuable reference.

### 3.3. Mitophagy Changes

Autophagy is a highly conserved biological process, which can be induced by growth factor deficiency, hypoxia, cell starvation, oxidative stress, and other conditions, and maintains cell survival and homeostasis by degrading and reusing some intracellular proteins and organelles.

Mitophagy is a process in which cells selectively degrade damaged mitochondria by acting on themselves to control the number and quality of mitochondria, mediate cell differentiation and metabolic reprogramming [116], and the most typical pathway is PINK1-Parkin-mediated ubiquitin-dependent mitophagy [117]. Under stress conditions, when the mitochondrial outer membrane is damaged, the transmembrane transport of PINK1 is blocked and retained on the outer membrane, which recruits and activates the ubiquitin ligase Parkin1; the latter constructs a polyubiquitinated modification chain on the mitochondrial outer membrane protein, recruits the adaptor protein, and then the adaptor protein binds to LC3 to form a double-membrane-wrapped autophagosome. Autophagosomes subsequently bind to lysosomes and damaged mitochondria are engulfed [118].

Retinal cells can also protect themselves through mitophagy in the environment of high glucose.

Many studies have shown that mitophagy is upregulated during DR [119]. Devi et al. found that Parkin accumulated in mitochondria of retinal Müller cell line rMC1 under high glucose induction, and then induced autophagy [120]. Mitophagy plays a protective role in DR, and notoginsenoside R1 (NGR1) can alleviate the damage of retinal Müller cells by further enhancing PINK1-Parkin-dependent mitophagy [121]. Mitophagy is also critical for the function and survival of cone cells under hyperglycemic conditions [122]. In retinal vascular endothelial cells, activating bile acid G-protein-coupled membrane receptor (TGR5) enhances mitophagy, thereby reducing endothelial dysfunction and slowing the progression of DR [123]. In retinal pigment epithelial cells, Sirt3, a deacetylase in mitochondria, can activate mitophagy through Foxo3a/PINK1-Parkin pathway and protect the retina in a high glucose environment [124]. Normal mitophagy is essential for the maintenance of mitochondrial oxidative phosphorylation and ATP synthesis, which may be one of the mechanisms for mitophagy to exert cytoprotective effects in DR [125].

Thioredoxin-interacting protein (TXNIP) plays an important role in the maintenance of cellular homeostasis in the pathological process of DR. Under high glucose stress, the expression of TXNIP increases, which can antagonize the oxidative stress [126]. On the other hand, TXNIP can also play a cytoprotective role in promoting mitochondrial division and autophagy [127]. Up-regulated TXNIP in high glucose induces nitroso-modification of dynamin related protein 1 (Drp1) to promote mitochondrial fission [128], while modified Drp1 promotes TXNIP translocation to mitochondria, mediating mitophagy through multiple pathways [129]. For example, TXNIP can promote LC3BII-mediated mitophagy in retinal Müller cells of diabetic rats [120]. TXNIP causes nitrosylation, nuclear export, and cytoplasm localization of high mobility group box 1 protein (HMGB1), which competes with the cytoplasm-localized anti-apoptotic protein B-cell lymphoma 2 (BCL-2) for binding to the autophagy-related protein Beclin 1 [130], thereby inducing autophagy.

However, some scholars have observed the inhibition of mitophagy pathway in DR. For example, Xie et al. observed that under a high glucose environment, the expression of voltage-dependent anion channel 1 (VDAC1) was significantly down-regulated in the mitochondrial membrane of human retinal capillary endothelial cells [131]. VDAC1 can recruit adaptor proteins such as OPTN and p62 after ubiquitination, thereby promoting Pink1-Parkin-mediated mitophagy [120].

In this regard, the highly accepted explanation is that the level of mitophagy depends on the degree of hyperglycemia. Zhang et al. observed in retinal pigment epithelium cultured in vitro that a slight increase in glucose concentration (15 mM) induced the upregulation of mitophagy, while a large increase in glucose concentration (50 mM) inhibited mitophagy, and the cells tended to apoptosis [132]. The corresponding mechanism may be that mild hyperglycemia induces stress response, which makes cells “self-protect” through mitophagy. Severe or persistent hyperglycemia can cause cell damage, resulting in mitophagy disorder [119].

### 3.4. Mitochondrial Pathway Apoptosis

As mentioned previously, oxidative stress is prone to occur in cells under high glucose conditions. Antioxidant substances in retinal cells of diabetic individuals are reduced (e.g., MnSOD is reduced [133,134]), while the oxidative system is hyperactive (e.g., Nox2 is increased [135]), and a large amount of ROS is generated to cause mitochondrial damage. Mitochondrial damage can induce apoptosis of retinal capillary endothelial cells, pericytes, and neurons through content extravasation and activation of apoptosis-related signaling pathways [136,137,138,139]. Among them, pericytes are more sensitive to high glucose and more prone to apoptosis than endothelial cells [140].

- Extravasation of proapoptotic substances

The opening of the PT pore in the outer mitochondrial membrane and the extravasation of proapoptotic substances are considered to be the characteristic events of mitochondrial pathway apoptosis. In retinal endothelial cells, high glucose can induce the release of mitochondrial cytochrome C, which may be related to the decrease of Cx43 channel activity in the outer mitochondrial membrane [104]. The release of cytochrome C triggers a caspase-mediated cascade that ultimately induces apoptosis. Santiago et al. found that high glucose-induced apoptosis in retinal neurons was related to the release of apoptosis-inducing factor (AIF) by mitochondria, but not dependent on the activation of caspases [141]. It is worth mentioning that RNA in mitochondria can also be released into the cytosol during mitochondrial damage induced by high glucose and exist as double-stranded RNA [142], which can interact with RNA-dependent protein kinase (PKR), mediating apoptosis of retinal neurons [143]. High glucose activates the NF-κB signaling pathway, enhances oxidative stress in Müller cells, and is also involved in inducing apoptosis in the mitochondrial pathway [144]. As a transcription factor, NF-kB can promote the expression of Matrix Metalloproteinase-2 (MMP-2) and MMP-9 [145], both of which are involved in inducing apoptosis of retinal microvascular endothelial cells [112,115,146]. In the diabetic state, MMP-2 is activated, which promotes the production of superoxide, accelerating mitochondrial membrane damage and cytochrome C leakage [146]. MMP-9 is also upregulated in high glucose and accumulates in mitochondria, increasing mitochondrial membrane permeability, which in turn promotes the entry of the pro-apoptotic protein BCL2-associated X protein (Bax) into mitochondria [112,115], mediating the occurrence of apoptosis.

- Disequilibrium of calcium homeostasis

High glucose can also induce apoptosis through the mitochondrial pathway by triggering disequilibrium of calcium homeostasis. Liu et al. found that sarcoplasmic/endoplasmic reticulum calcium ATPase 2 (SERCA2) is inactivated by irreversible oxidative modification of Cys674 residue, which disrupts intracellular calcium homeostasis and promotes apoptosis [147,148].

- Mitochondrial dynamic imbalance

In addition, the dynamic imbalance of mitochondrial fusion and fission is also closely related to apoptosis. High glucose stimulation can change the activity of related enzymes, reducing the fusion process of mitochondria in cells, and increasing the division, eventually stimulating the release of cytochrome C, and causing apoptosis of retinal cells (such as vascular endothelial cells and Müller cells) [149]. At the molecular level, high glucose can down-regulate the expression of mitochondrial fusion protein Mfn2 and fusion-related optic atrophy protein 1 (OPA1) in human retinal capillary endothelial cells [131,150], resulting in the reduction of mitochondrial fusion [151]. High glucose can also promote the phosphorylation and activation of PKCδ as well as upregulate TXNIP, regulating the phosphorylation and nitroso modification of a motility related protein Drp1, respectively, thus activating Drp1, which is related to mitochondrial fission [128,129]. Overexpression of Mfn2 or knockdown of Drp1 can inhibit apoptosis of retinal endothelial cells induced by high glucose [152,153,154].

## 4. Conclusions and Future Perspectives

As mentioned above, under the condition of high glucose, retinal cells will undergo reprogramming of glucose metabolism, including overactivation of the polyol pathway and hexosamine pathway, while generating AGEs to mediate a variety of pathological changes. Excessive lipid accumulation and the production of abnormal lipid metabolites, such as ceramide and acrolein, also exert damaging effects. The hypermethylation of nuclear-encoded mitochondrial function-related genes and mitochondrial genome genes under high glucose stress promotes the expression of oxidative stress related genes, while inhibiting the expression of antioxidant genes and mtDNA repair genes. Many factors can cause mitochondrial dysfunction and eventually induce mitochondrial pathway apoptosis. In addition, high glucose stress can also affect mitophagy, and affect cell activity by disrupting the balance of mitochondrial fusion and division. These mitochondria-related mechanisms may play an important role in the pathogenesis of DR.

Systemic treatment of diabetes to stabilize blood glucose levels is undoubtedly the most effective way to prevent DR or delay its progression [155]. In view of the above mechanisms of high glucose-induced retinopathy, people have begun to explore the possibility of treatment by inhibiting these mitochondria-related pathophysiological processes.

Oxidative stress induced by metabolic disorder in a high glucose environment is the most critical factor that causes mitochondrial damage and even induces mitochondrial pathway apoptosis. Therefore, scavenging ROS, inhibiting their production, or enhancing the antioxidant capacity of cells are the most commonly strategies. In order to delay the progression of DR, scientists have carried out many animal experiments and clinical studies, and have obtained some encouraging outcomes [156]. Many ROS scavengers and antioxidants, such as ubiquinone, lipoic acid, taurine, polyphenols, and trace elements such as zinc, manganese and selenium, have been proved to be able to improve the mitochondrial function of retinal ganglion cells and pigment epithelial cells, reduce hypoxia-induced angiogenesis, and thus slow down the progress of DR [30,157,158,159,160,161,162]. Exogenous L-carnitine and plant flavonoid quercetin can promote the expression of antioxidant substances such as GSH, SOD and catalase in the retina of diabetic rats, exerting anti-apoptotic effect and preventing diabetic retinal neurodegeneration [163,164]. Fenofibrate and simvastatin can inhibit retinal endothelial cell apoptosis and pericyte loss by targeting peroxisome proliferator-activated receptor (PPAR) and its coactivators to inhibit ROS production. It has a protective effect on retinal blood vessels under a high glucose environment [165,166,167].

Other drugs or methods against mitochondrial pathway apoptosis have also been studied in the prevention and treatment of DR. MTP-131 and 17β-estradiol can stabilize the mitochondrial membrane, inhibit the release of pro-apoptotic factors such as cytochrome C, Bax, and the production of cleaved-caspase-3, and significantly reduce the apoptosis of retinal ganglion cells [168,169]. Epigallocatechin-3-gallate (EGCG), a major component of tea polyphenols, can stimulate the autophagy of retinal Müller cells, resulting in reactive glial hyperplasia and having an anti-apoptotic effect [170], as well as inhibiting the NF-κB signaling pathway and reducing the formation of MMP-9, thereby exerting an anti-apoptotic effect (see Section 3.4, Mitochondrial Pathway Apoptosis) [171]. Human 8-OXoG DNA glycosylase/apurinic lyase (hOGG1) plays a protective role in mitochondria by promoting the repair of damaged mtDNA [172].

As mentioned earlier (see Section 3.4, Mitochondrial Pathway Apoptosis), the reduction of mitochondrial fusion or increased mitochondrial fission is closely related to apoptosis, and the imbalance dynamics of mitochondria also plays an important role in the DR process. As a result, drugs that inhibit excessive mitochondrial division have attracted attention in recent years, including mitochondrial division inhibitor-1 (mdivi-1), dynasore, P110, 15-oxospironolactone, and tanshinone, all of which are likely to have protective effects on mitochondria in a high glucose environment [173,174]. Their protective effect on the retina of diabetic individuals needs to be further explored. Melatonin can maintain mitochondrial homeostasis and protect the blood-retinal barrier by down-regulating the expression of genes related to mitochondrial fission (such as DRP1, hFis1, MIEF2, MFF) and up-regulating the expression of genes related to mitochondrial biogenesis (such as PGC-1α, NRF2, PPAR-γ), thus inhibiting the development of diabetic macular edema [175]. Serum copper level is increased in DR patients, which is associated with abnormal mitochondrial morphology. The copper chelator penicillamine increases the level of the mitochondrial fusion protein mfn2, thereby restoring normal mitochondrial morphology and function [176]. A summary of drugs and means targeting mitochondria to treat DR is presented in Table 1.

Regarding mitochondrial dynamic homeostasis, in addition to mitophagy, fusion and fission, recently, scholars have begun to focus on mitochondrial extrusion and intercellular transfer in many fields such as stroke, cardiovascular disease, and cancer [206]. On one hand, damaged or senescent mitochondria can be extruded from cells to maintain cellular homeostasis and prevent apoptosis. This can occur under both physiological and pathological conditions. Retinal ganglion cell axons can shed mitochondria at the optic nerve head, which are internalized and degraded by adjacent astrocytes [207]. Mitochondrial transfer between retinal photoreceptor cells can also occur through intercellular tunneling nanotubes (TNTs), which ensures the normal metabolism and function of cells under light stimulation [208]. Under the stress of ischemia and reperfusion, the damaged mitochondria (decreased membrane potential and ATP synthesis ability) can be extruded, which may serve as a quality-control mechanism to ensure cellular homeostasis [209]. Mitochondria are damaged under high glucose conditions, and it has not been revealed whether a similar phenomenon also exists in retinal cells as an adaptive protective mechanism. On the other hand, exogenous mitochondrial transplantation can rescue the activity of damaged cells in many disease models, and has potential therapeutic value in neurodegenerative diseases, tumors, myocardial injury and other diseases [210,211,212]. Therefore, whether mitochondrial transplantation can improve the activity of damaged cells to delay the progression of DR is also worth exploring.

## Figures and Tables

**Table 1:** Drugs and means targeting mitochondria to treat DR.

Drugs or Means	Mitochondria Related Mechanisms and Targets	Effect in DR	Experimental Objects and Methods
Ubiquinone [161]	Promote electron transfer and ATP synthesis in mitochondrial respiratory chain	Improve mitochondrial function of platelets in patients with NPDR	Clinical trials
Short-Chain Quinones [177]	Antioxidant effect	Inhibit retinal ganglion cell loss, reactive glial hyperplasia, vascular leakage and retinal thinning	Animal Experiment (rat)
Zeaxanthin [178]	Inhibit the formation of lipid peroxides, maintain the activity of complex III and the level of mitochondrial SOD	Inhibit oxidative stress of retina	Animal Experiment (rat)
Lactucaxanthin [179]	Resist oxidation and restore mitochondrial membrane potential	Prevent the damage of RPE cells	Cell Experiment (ARPE-19)
Lutein [180,181]	Resist oxidation, restore mitochondrial membrane potential, enhance AMPK phosphorylation, and upregulate the expression of PGC-1α, NRF1 and TFAM, so as to maintain the integrity of mtDNA and normal mitochondrial biogenesis	Prevent the damage of RPE cells	Cell Experiment (ARPE-19) Animal Experiment (rat)
Lipoic acid [160,162]	Resist oxidation and prevent the formation of ROS	Prevent damage to microvessels and pericytes	Animal Experiment (rat)
*N*-acetylcysteine (+SS31,mitochondrial antioxidant) [182,183,184]	Enhance the interaction between cytochrome c, facilitate better electron transfer from complex III to complex IV, relieve mitochondrial dysfunction, oxidative stress and reduce mitophagic flux to lysosomes	Prevent the damage of RPE cells	Cell Experiment (ARPE-19)
Edaravone [185]	Clear ROS and restore mitochondrial membrane potential	Protect retinal ganglion cells	Cell Experiment (Primary Müller cell)
U83836E [186]	Restore the level of mitochondrial SOD and maintain the normal morphology of mitochondria	Improve the electrophysiological function of retinal ganglion cells, thereby relieving neurodegeneration in DR	Animal Experiment (rat)
Hydrogen sulfide donor GYY4137 [187]	Relieve oxidative stress, reduce MMP-9, and maintain mitochondrial integrity	Inhibit the apoptosis of RECs	Animal Experiment (mouse) Cell Experiment (HREC)
AMPK agonists (e.g., metformin) [188]	Restore mitochondrial membrane potential and abnormal morphology	Delay photoreceptor degeneration caused by diabetes	Animal Experiment (mouse)
Wnt inhibitory factor 1 [189]	Downregulate AMPK/mTOR pathway, improve mitochondrial function, restore mitochondrial membrane potential, and resist oxidation	Inhibit neovascularization and protect RPE cells	Cell Experiment (ARPE-19) Animal Experiment (mouse)
670 nm photobiomodulation [190,191]	Increase the membrane potential and maintain the integrity of photoreceptor mitochondria	Protect Müller cells and photoreceptors from damage	Animal Experiment (mouse) Cell Experiment (rat Müller cell) Clinical trials
miR-451a [192]	Target ATF2 on the outer membrane of mitochondria to stabilize mitochondrial membrane potential and respiratory function	Inhibit the abnormal proliferation and migration of RPE cells in PDR	Cell Experiment (293T, ARPE-19)
Genipin [193]	Promote AKT signal pathway and regulate miR-4429/JAK2 signal axis	Maintain normal metabolism and membrane stability of mitochondria	Cell Experiment (APRE-19)
Notoginsenoside R1, NGR1 [121]	Enhance PINK1/Parkin dependent mitophagy	Alleviate the damage of Müller cells	Cell Experiment (rMC-1)
Taurine [194]	Reduce the expression of mitochondrial dependent apoptosis genes	Prevent retina cells from apoptosis	Animal Experiment (rat)
Polyphenols [159,170,195]	Inhibit the production of mitochondrial ROS and the expression of mitochondrial related pro-apoptotic factors; Adjust mTOR pathway	Inhibit the oxidative damage and apoptosis of optic nerve cells; Inhibit the formation of acellular capillaries and pericyte ghost; Promote autophagy and inhibit the apoptosis of Müller cells	Animal Experiment (mouse, rat) Cell Experiment (Primary rat Müller cell)
L-carnitine [163]	Reverse the change of mitochondrial membrane potential and the release of Cyt c; inhibit ROS production and lipid peroxidation; down-regulate apoptosis related proteins	Inhibit the apoptosis of retinal ganglion cells	Cell Experiment (Primary rat RGC)
Quercetin [196]	Promote the expression of antioxidant enzymes and inhibit the expression of mitochondrial related pro-apoptosis factors	Prevent diabetic retinal neurodegeneration and oxidative stress injury	Animal Experiment (rat)
Fenofibrate [165,167]	PPAR-α Agonists, inhibit mitochondrial ROS production	Inhibit the apoptosis of retinal vascular endothelial cells and pericyte loss	Cell Experiment (RF/6A) Animal Experiment (mouse)
hOGG1 [172]	Promote the repair of mtDNA damage	Inhibit the apoptosis of RECs	Cell Experiment (rREC)
GSK-3β inhibitor [197,198]	Inhibit the hyperphosphorylation of tau protein and maintain the normal transport and function of mitochondria in nerve cells	Regulate the apoptosis of retinal glial cells and inhibit synaptic neurodegeneration in early DR	Cell Experiment (Primary rat neurons and glial cells)
Hu-Zhang-Qing-Mai-Yin [199]	Increase p-P38 and ROS, decrease ATP level, and downregulate the expression of BCL-XL and BCL-2	Promote mitochondrial apoptotic pathway of RECs	Cell Experiment (HRECs)
Astragalus Polysaccharide [200,201]	Adjust miR-182/Bcl-2 axis and miR-195/Bcl-2 axis to alleviate mitochondrial damage	Inhibit the apoptosis of RPEs	Cell Experiment (ARPE-19)
MTP-131 [169]	A novel mitochondrial targeting peptide that inhibits H_2_O_2_ induced mitochondrial damage and cytochrome c release	Prevent retinal ganglion cells from apoptosis	Cell Experiment (RGC-5)
E2 [168]	Stabilize mitochondrial membrane potential, reduce intracellular ROS level, up regulate Bcl-2 expression, inhibit Bax expression, and reduce the leakage of cytochrome c	Prevent retinal ganglion cells from apoptosis	Cell Experiment (RGC-5)
Exendin-4 [202]	Resist oxidation, downregulate NADPH, inhibit c-Jun *N*-terminal kinase, and downregulate protein kinase-β and p66Shc	Prevent mitochondrial apoptotic pathway of RPEs	Cell Experiment (ARPE-19)
TSHR-siRNA [203]	Block TSH receptors in retinal microvascular pericytes	Prevent mitochondrial apoptotic pathway of retinal pericytes	Cell Experiment (HRMVPCs)
Prohibitin (PHB) [204]	Inhibit ROS production and maintain mitochondrial homeostasis	Prevent RECs from apoptosis	Cell Experiment (HRECs)
Dixipamine [68]	Inhibit acid sphingomyelinase, inhibit the production of ceramide, and restore the normal metabolism of mitochondria	Restore normal function of RPEs	Animal Experiment (rat)
Simvastatin [166]	Upregulate PGC-1α and inhibit mitochondrial ROS/PARP pathway	Inhibit retinal vascular damage in early stage of diabetes	Animal Experiment (rat)
Tanshinone IIa [173]	Inhibit excessive fission of mitochondria and increase mRNA levels of mfn1 and opa1	Inhibit methylglyoxal-induced injury of RECs	Cell Experiment (BREC)
Melatonin [175]	Downregulate the expression of mitochondrial fission related genes and upregulate the expression of mitochondrial biogensis related genes	Inhibit the apoptosis of RPEs and treat DME	Cell Experiment (ARPE-19)
Mdivi-1 [129]	Suppress PKC δ/Drp1 signal pathway and prevent excessive fission of mitochondria	Reverse retinal vascular leakage, acellular capillary formation and apoptosis	Cell Experiment (HRECs)
TGR5 [123]	Suppress PKC δ/Drp1 signal pathway and prevent excessive fission of mitochondria; regulate PINK1/Parkin signal pathway and enhance mitophagy	Inhibit the apoptosis of RECs	Cell Experiment (HREC)
Overexpress SIRT3 [124]	Activate mitophagy via Foxo3a/PINK1-Parkin pathway	Inhibit the apoptosis of RPEs	Cell Experiment (ARPE-19)
Penicillamine [176]	Chelate copper ions and increase the level of mitochondrial fusion protein mfn2	Increase the activity of retinal pigment cells; inhibit ER stress and inflammation	Cell Experiment (ARPE-19)
SNGH16 [205]	Target miR-195 to increase the protein level of mfn2	Inhibit pathological angiogenesis	Cell Experiment (HRECs)
